# Sender Gender Influences Emoji Interpretation in Text Messages

**DOI:** 10.3389/fpsyg.2019.00784

**Published:** 2019-04-05

**Authors:** Sarah E. Butterworth, Traci A. Giuliano, Justin White, Lizette Cantu, Kyle C. Fraser

**Affiliations:** Department of Psychology, Southwestern University, Georgetown, TX, United States

**Keywords:** emoji, emoticons, perceptions, gender, gender differences, text messages, texting

## Abstract

With the rise in social media use, emojis have become a popular addition to text-based communication. The sudden increase in the number and variety of emojis used raises questions about how individuals interpret messages containing emojis. To explore perceptions of emoji usage, we conducted a 2 (Sender Gender: Female or Male) × 2 (Emoji Type: Affectionate or Friendly) between-groups experiment to examine the appropriateness and likability of each of four hypothetical text messages sent to a woman from either a male or female coworker. In general, we predicted that text messages containing affectionate emojis (i.e., kissing-face and heart emoji) would be perceived as more appropriate and likable when they came from female than from male senders, whereas messages containing less overtly affectionate (but still friendly) emojis (i.e., smiling-face emoji) would be considered equally appropriate and likable whether it came from female or male senders. As predicted, the results confirmed that texts with affectionate emojis were judged as more appropriate and likable when they came from women than from men. However, texts with less affectionate but friendly emojis were judged as equally appropriate–but more likable–when they came from men than when they came from women. Taken together, our results indicate that gender and emoji choice influence perceptions, and therefore people should consider how emoji choice could impact the reception of their message.

## Introduction

Emojis are pictographs used in digital communication that can provide an emotional context to text-based communication, potentially strengthening the impact, comprehension, and interpretation of a message by accentuating its intended positivity, negativity, or neutrality (Derks et al., 2008; Lo, 2008; Skovholt et al., 2014; Dunlap et al., 2016; Kaye et al., 2016; Miller et al., 2016). However, the interpretation of emojis can also be ambiguous (Rodrigues, 2018). Variations in the perceived meaning and emotionality of emojis can be due to the platform on which the emoji appears (Miller et al., 2016), the intended emotionality of the message (Derks et al., 2008), characteristics of individuals rating the emoji (Byron and Baldridge, 2007; Novak et al., 2015), and personality traits that have been associated with specific emoji choice (Marengo et al., 2017; Li et al., 2018).

An important attribute that could potentially explain differences in message and emoji interpretation is the gender of the sender of the message. For example, women use twice as many emojis compared to men (Tossell et al., 2012) and use them in different ways (e.g., for emotional aims; Wolf, 2000; Fullwood et al., 2013). These differences, combined with expectations and stereotypes about gendered communication (e.g., that women are expected to be more relationship-oriented, understanding, and supportive; Felmlee et al., 2012; Iafrate et al., 2012), lead people to perceive female messengers as more adept at virtual conversation than male messengers (Ling et al., 2014). Given that stereotypical gender expectations might bias receivers’ interpretations about senders, receivers may interpret a message differently because they expect to encounter a greater number of emojis and more emotionally inflected usage of emojis when interacting with females than with males (Wolf, 2000; Fullwood et al., 2013; Ling et al., 2014).

Not only are there differences in the number and type of emojis used by men and women (Tossell et al., 2012; Ling et al., 2014), but there are also perhaps differences in female and male motivation for using certain emojis. For instance, previous research on relationships between emoji and personality type has shown that certain emojis (such as those considered affectionate), are closely correlated with agreeableness, a predominately female trait (Weisberg et al., 2011; Li et al., 2018). In contrast, friendly, smiley-faced emojis may be preferred by people who are high in extraversion, a trait that splits more complexly along gender lines (Weisberg et al., 2011; Marengo et al., 2017; Li et al., 2018). That is, subcategories of extraversion (e.g., warmth vs. assertiveness) differ between men and women, making extraversion a less obviously gendered trait than agreeableness (Weisberg et al., 2011). As such, the findings of Weisberg et al. (2011) suggest that it would be more acceptable for women to use the affectionate and agreeable emojis than it would be for men. Alternatively, friendly and extraverted smiling-face emojis may represent a more gender-neutral option for men because the personality traits associated with these types of emojis are not as clearly gendered.

Given that gender differences may play a role in the interpretation of emojis, the present study investigated the extent to which the gender of a message’s sender and the sender’s choice of emojis impact the perceptions of messages. Specifically, we manipulated the gender of a sender of a hypothetical text message (i.e., a text to a female coworker or a male coworker), as well as the types of emojis contained in the message (i.e., affectionate emojis or friendly emojis), and asked participants to rate the appropriateness of the message and the likability of the sender. The text messages between coworkers included either (a) a kissing-face and red heart emoji, designed to communicate affection, or (b) a smiling-face emoji, intended to communicate friendliness. Given common societal gender stereotypes about communication, namely that women are expected to be more communicative, agreeable, and apt to use emojis relative to men (e.g., Wolf, 2000; Weisberg et al., 2011; Ling et al., 2014), we made two predictions:

(H1) A text message containing affectionate emojis would be perceived as more appropriate and likable when it came from a female sender rather than a male sender, and
(H2) A text message containing an emoji that was less overtly affectionate but still friendly would be considered equally appropriate and likable whether it came from a female sender or a male sender.

## Materials and Methods

### Participants and Design

Eighty undergraduate students (40 men, 39 women, and 1 unreported) from a small liberal arts college in central Texas voluntarily participated in the present study for no compensation. They ranged in age from 18 to 30 years (*M* = 20.14; *SD* = 1.87) and self-reported their ethnicities as White (47.5%), Hispanic (27.5%), Asian (12.5%), Black (1.3%), Multi-racial (6.3%), or *Other* (3.8%), with 1.3% unreported. Participants were recruited through convenience sampling at various locations on campus and asked if they would be willing to complete a 5-min survey on “workplace correspondence.” This study was reviewed and approved by Southwestern University’s Institutional Review Board, and all subjects gave written informed consent in accordance with the Declaration of Helsinki.

The text message manipulation consisted of a screen shot of a hypothetical iMessage in which one coworker thanked the other for a work-related favor (“Hey Katie, I’m sorry I couldn’t come in yesterday. I’m feeling a lot better today though. Thanks for covering my shift”). As part of a 2 × 2 between-subjects design, the text message (which was always addressed to a female coworker) came from either a female (“Rebecca”) or a male (“Steven”) sender, and contained either an affectionate combination of emojis (i.e., kissing-face emoji and red heart emoji) or a less overtly affectionate, but still friendly, emoji (i.e., grinning smiley face). See Appendix for the four text message conditions.

### Procedure and Measures

After reading the text message, participants were asked to make judgments about the sender and the message on 7-point Likert scales ranging from 1 (*Strongly Disagree*) to 7 (*Strongly Agree*). First, participants rated the appropriateness of the message (six items; α = 0.89) on the following items: “I would send a message like this to a coworker,” “I find this text message acceptable,” “[Rebecca/Steven] acted professionally in this case,” “The text shows a proper tone between employees,” and two reverse-coded items, “Katie probably felt uncomfortable receiving this text,” and “This message seems inappropriate.” Next, participants rated the likability of the sender (four items; α = 0.88): “I would like to work with someone like [Rebecca/Steven],” “I feel as though I would get along well with [Rebecca/Steven],” “[Rebecca/Steven] seems like the kind of person who gets along well with others,” and “[Rebecca/Steven] is probably a likable person.”

Pilot tests were used to verify that the kissing-face emoji was perceived as significantly more affectionate (*M* = 8.73 out of 10) than the smiling-face emoji (*M* = 3.82), *t*(10) = 7.36, *p* < 0.001, and that the photographs of the male and female senders (used to increase the salience of their gender) were perceived as equally attractive (both *M*s = 6.91 out of 10), *t*(10) = 0.00, *ns*. Because early pre-tests of participants with the actual experimental materials revealed that some participants mistakenly perceived the kissing-face emoji as the smiling-face emoji, we added the heart emoji immediately following the kissing-face emoji to the *affectionate emoji* text message in order to increase the salience and impact of the manipulation in that condition.

## Results

A pair of 2 (Sender Gender) × 2 (Emoji Type) between-subject analyses of variance (ANOVAs) was used to analyze the effects of the gender of the message sender and emoji affectionateness on the perceived appropriateness of the message and likability of the sender. Notably, a 2 × 2 × 2 ANOVA with participant gender did not moderate the pattern of results; that is, there were no significant 3-way (*F*s < 1) or 2-way (*F*s < 1.29, *p*s > 0.26) interactions involving participant gender for either dependent variable, and thus participant gender is not discussed further.

**FIGURE 1:**
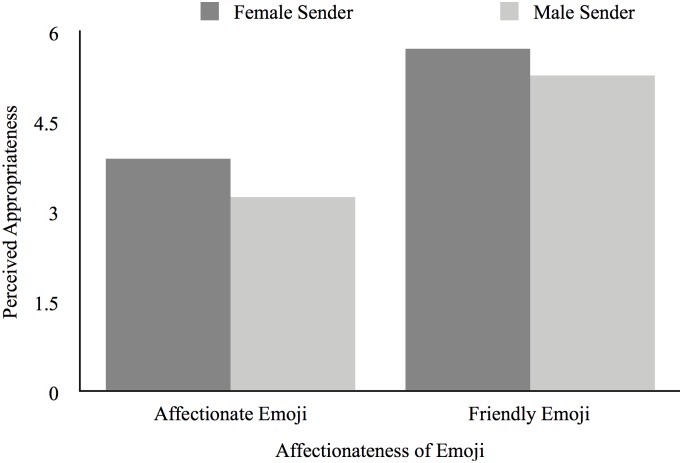
Perceived appropriateness of text as a function of the sender’s gender and emoji choice.

The results revealed a main effect of emoji type on perceived appropriateness, such that texts that contained affectionate emojis (*M* = 3.57; *SD* = 1.01) were generally perceived as less appropriate than were texts that contained a less affectionate, but friendly, emoji (*M* = 5.50; *SD* = 0.85), *F*(1,76) = 91.66, *p* < 0.001, $n_{p}^{2}$ = 0.55. As expected, this main effect was qualified by a significant interaction between gender of sender and type of emoji, *F*(1,76) = 6.98, *p* = 0.01, $n_{p}^{2}$ = 0.08. As Figure 1 shows, when a text message containing an affectionate emoji was sent by a man (*M* = 3.25; *SD* = 0.97), it was perceived as less appropriate than when it was sent by a woman (*M* = 3.89; *SD* = 0.97), *t*(38) = 2.10, *p* = 0.04, *d* = 0.68. However, when a text message containing a less-affectionate but friendly emoji was sent by a man (*M* = 5.72; *SD* = 0.78), it was perceived as equally appropriate as when it was sent by a woman (*M* = 5.29; *SD* = 0.89), *t*(38) = -1.61,*p* = 0.12, *d* = -0.52.

**FIGURE 2:**
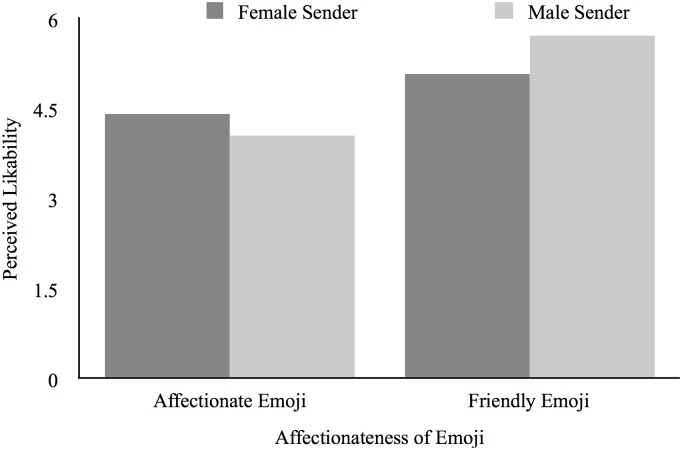
Text sender’s perceived likability as a function of the sender’s gender and emoji choice.

There was a similar main effect of emoji type on sender’s perceived likability, *F*(1,76) = 30.08, *p* < 0.001, $n_{p}^{2}$ = 0.28, revealing that senders of text messages containing affectionate emojis (*M* = 4.24; *SD* = 1.00) were perceived as less likable than were senders of text messages containing less affectionate but friendly emojis (*M* = 5.40; *SD* = 0.93). As shown in Figure 2, this effect was again qualified by the predicted interaction between sender gender and emoji type, *F*(1,76) = 5.48, *p* = 0.02, $n_{p}^{2}$ = 0.07. That is, when a message containing an affectionate emoji was sent by a man (*M* = 4.06; *SD* = 0.96), the sender was perceived as equally likable as when the text message was sent by a woman (*M* = 4.43; *SD* = 1.04), *t*(38) = 1.15, *p* = 0.26, *d* = 0.37. Unexpectedly, when a message containing a less-affectionate but friendly emoji was sent by a man (*M* = 5.71; *SD* = 0.69), the sender was perceived as more likable than when the message was sent by a woman (*M* = 5.09; *SD* = 1.05), *t*(32.89) = -2.23, *p* = 0.03, *d* = -0.78. There were no main effects of sender gender on either perceived appropriateness of the message or likability of the sender (both *F*s < 1, *ns*), indicating that male and female senders were perceived as equally appropriate and likable.

## Discussion

Our findings confirm that people’s perceptions of a message and its sender can be affected not only by the gender of the sender, but also by his or her emoji usage. These findings are consistent with gender stereotypes in communication (e.g., Sullins, 1992; Weisberg et al., 2011; Ling et al., 2014), showing that people generally perceive affectionately emotive women as more appropriate than affectionately emotive men. Because men are not expected to show as much emotion in their communication, however, when they do show emotion they may potentially receive more of a positive reaction than women do (Witmer and Katzman, 1997; Wolf, 2000; Tossell et al., 2012), as did the male coworker in the present study, who used the friendly but not-overly affectionate emoji. The ratings of the senders of the friendly emojis do not by any means demonstrate that women are disliked for their usage of friendly emojis, but rather that men are perhaps more celebrated for acting in a way that is inherently more expected in female communication (Tossell et al., 2012). Taken together, our findings indicate that stereotypes and expectations about communication can ultimately shape our perceptions of others.

In this exploratory first study, we focused primarily on two types of emojis: affectionate and friendly. In the future, replication with a variety of emojis would be important to extend the generalizability of our results. In the current study, the smiling-face emoji was used to represent the *less affectionate but friendly* condition, whereas a combination of the kissing-face and heart emoji (to ensure that participants noticed the kissing-face) was used to represent the *affectionate emoji* condition. Admittedly, the different number of emojis between conditions could provide a possible alternative explanation to differences in judgements, but based on prior research, it seems likely that the kissing-face and red heart emojis both represent affection, liking, and loving (Li et al., 2018). Therefore, the kissing-face and red heart emojis should serve to communicate (and strengthen) the same intended message for the affectionate condition. Although future studies will benefit from exploring a more diverse range of emojis, our study contributes to the empirical understanding of gendered judgements of emoji use in text-based communication.

Although the relatively homogenous nature of our college-student sample limits the generalizability of our results, the door has been opened to future research including a more diverse sample investigating messages sent to both female and male recipients. Because people have a tendency to make heteronormative assumptions (e.g., Simoni and Walters, 2001), the male sender may have been perceived as more flirtatious and less appropriate than the female sender simply because the hypothetical receiver of the message was female and thus may have been perceived by participants as a target of romantic interest by the male coworker. Future research could remedy this potential confound by either manipulating both the gender or the sender and receiver (to examine all sender/receiver gender combinations) or by using a hypothetical receiver with a gender-neutral name in order to examine judgements of appropriateness in a way that minimizes the inference of potential romantic interest.

Given research showing that emoji usage occurs less frequently in task-oriented interactions than in social interactions (Derks et al., 2007), the use of emojis in workplace communication merits further study to more thoroughly understand workplace dynamics. In the present study, it seems plausible that gendered constraints and expectations may provide a potential explanation for why “Steven” was perceived as more likable for his less affectionate-but-friendly emoji use compared to “Rebecca.” That is, women who are perceived as competent in the workplace can face a “backlash” for counter-stereotypical behavior (Rudman, 1998; Rudman et al., 2012). Hence, participants might have perceived “Rebecca” as more career-oriented and less relationship oriented, resulting in the backlash of lower likability compared to “Steven,” who was perceived as more likable by communicating in a friendly but not affectionate manner. Future research could also vary coworker status (e.g., boss/employee) and emoji usage of a message to explore the extent to which certain power dynamics or need for formality (Glikson et al., 2017) interact with gender to influence perceptions of emoji appropriateness in the workplace.

Our findings also highlight opportunities for further research on the effects of emoji use in general as well as on the interplay among personality, gender, and emoji use. For example, investigators could examine differences in perceived appropriateness as a function of use of an affectionate emoji, a friendly emoji, and no emoji at all, to understand the extent to which emoji use in general is influenced by sender gender, and in which specific contexts. Additionally, given the present results (as well as past research demonstrating that the perceptions of a message’s sender are largely based on the perceiver’s own personality; Byron and Baldridge, 2007), it would be helpful to examine the effects of individual differences on perceptions of emoji usage (e.g., personal emoji usage, self-disclosure tendency, and core personality traits) and the extent to which these variables moderate individuals’ perceptions of another’s message. It would be similarly beneficial to consider the potential influence of the sender’s personality in determining others’ perceptions of emoji use; for example, an extension of the current study could determine whether the perceived agreeableness and extraversion represented by the sender’s choice of emojis influence the perceptions of those emojis (Marengo et al., 2017; Li et al., 2018).

In addition to offering many avenues for future research, our results also suggest some practical applications. First, our findings indicate that it is important to be conscious of emoji selection in personal communication because emoji choice can affect perceptions of the message as well as perceptions of the senders themselves, consistent with past research (e.g., Walther and D’Addario, 2001; Ganster et al., 2012; Wang et al., 2014; Grieve et al., 2019). Moreover, our results highlight the benefits of using less-affectionate but friendly emojis in social communication. Specifically, in breaking the stereotype (e.g., Ling et al., 2014) and using emojis to accentuate emotional content, men could be perceived as surprisingly friendly and likable in their communications.

In closing, the present study offers a promising first step toward understanding the effects of gender and emoji usage on interpersonal perceptions. The implication that positive self-presentation can be either aided or hindered by a mere pictorial representation in a message is an important one. Indeed, because perceptions of a sender (based on his or her emoji use) can affect that sender’s future interactions, relationships, and opportunities–together with the increasing popularity of emojis–there exists a growing need for a better understanding of emojis and their effects.

## Ethics Statement

This study was carried out in accordance with the recommendations of Southwestern University’s Institutional Review Board. All subjects gave written informed consent in accordance with the Declaration of Helsinki.

## Author Contributions

SB contributed conception of the study, wrote the first draft and subsequent revisions, contributed to study design and materials, collected data, and conducted statistical analysis. TG edited the first draft and subsequent revisions, contributed to study design, edited the materials, and conducted statistical analyses. JW contributed to study design and materials, collected data, conducted statistical analysis, and edited revisions. LC and KF contributed to study design and materials, collected data, and conducted statistical analysis. All authors read and approved the submitted manuscript.

## Conflict of Interest Statement

The authors declare that the research was conducted in the absence of any commercial or financial relationships that could be construed as a potential conflict of interest.
