# Supplementary material for: Sender Gender Influences Emoji Interpretation in Text Messages
Source: Front Psychol. 2019 Apr 5;10:784. doi: 10.3389/fpsyg.2019.00784 (PMC6459937; doi:10.3389/fpsyg.2019.00784)

Appendix

Experimental Manipulation: Female Sender and Non-Affectionate Emoji

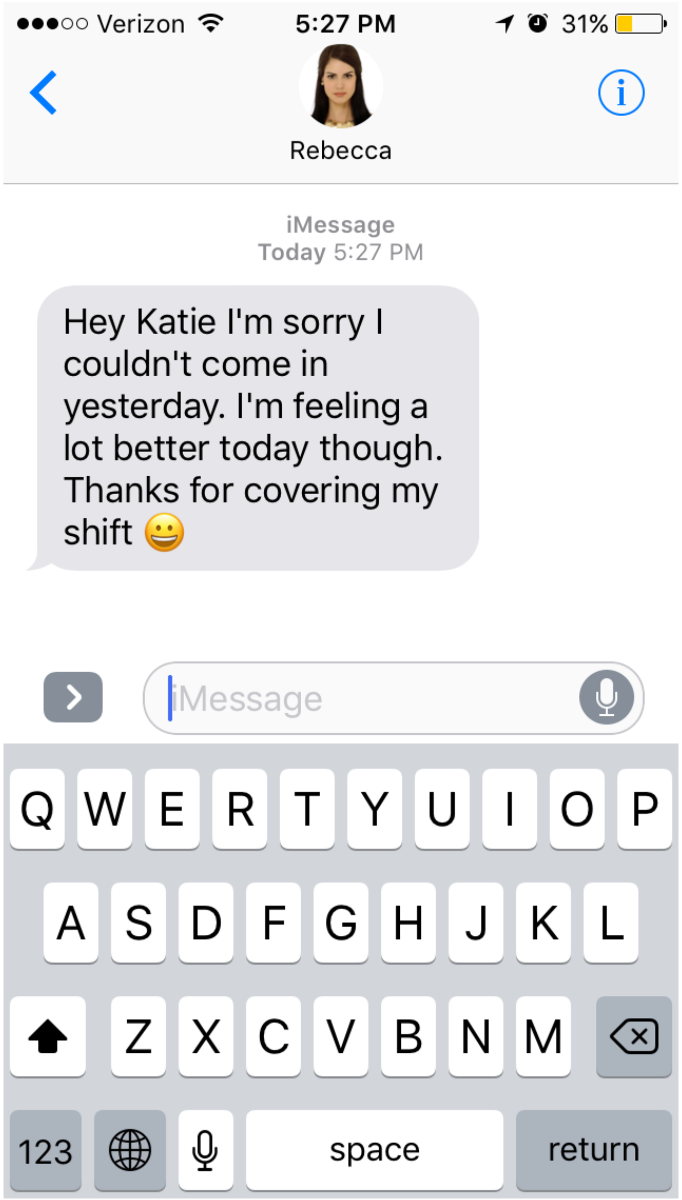

Experimental Manipulation: Male Sender and Non-Affectionate Emoji

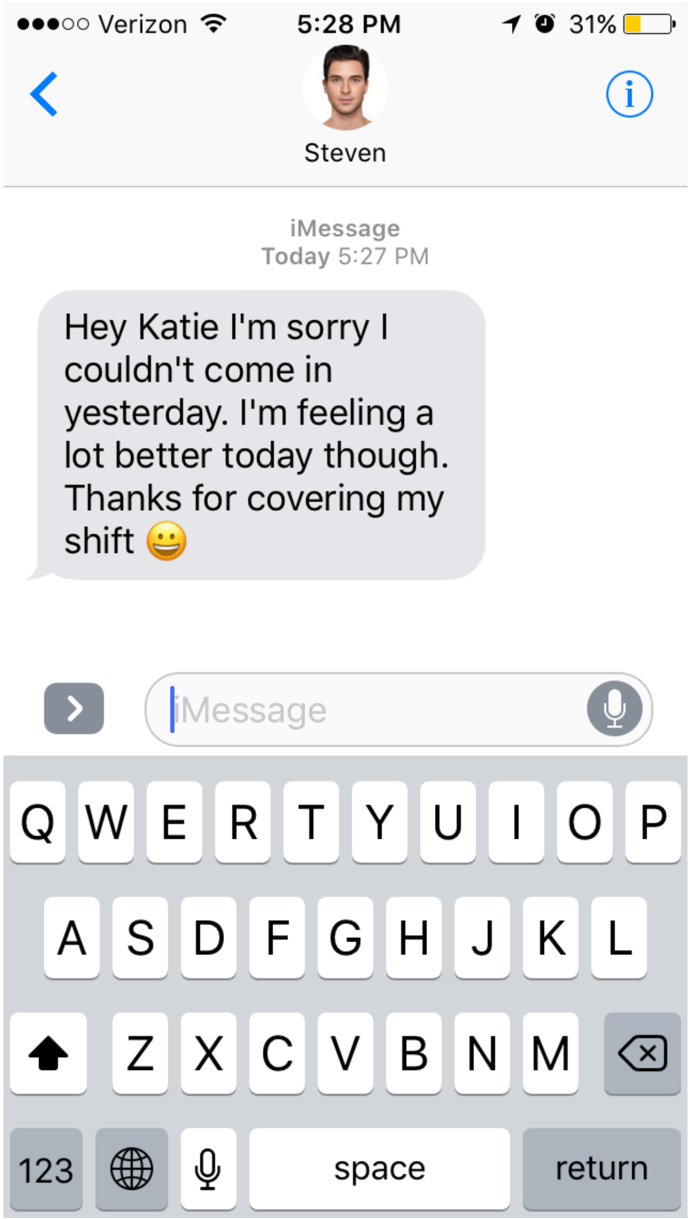

Experimental Manipulation: Female Sender and Affectionate Emoji

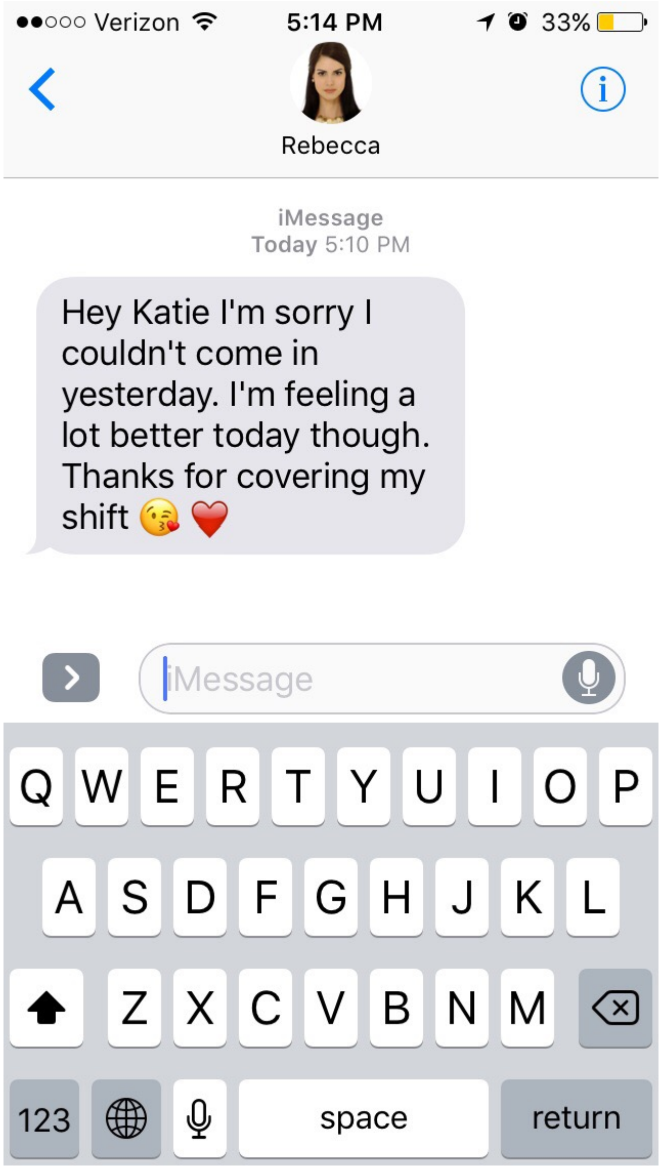

Experimental Manipulation: Male Sender and Affectionate Emoji

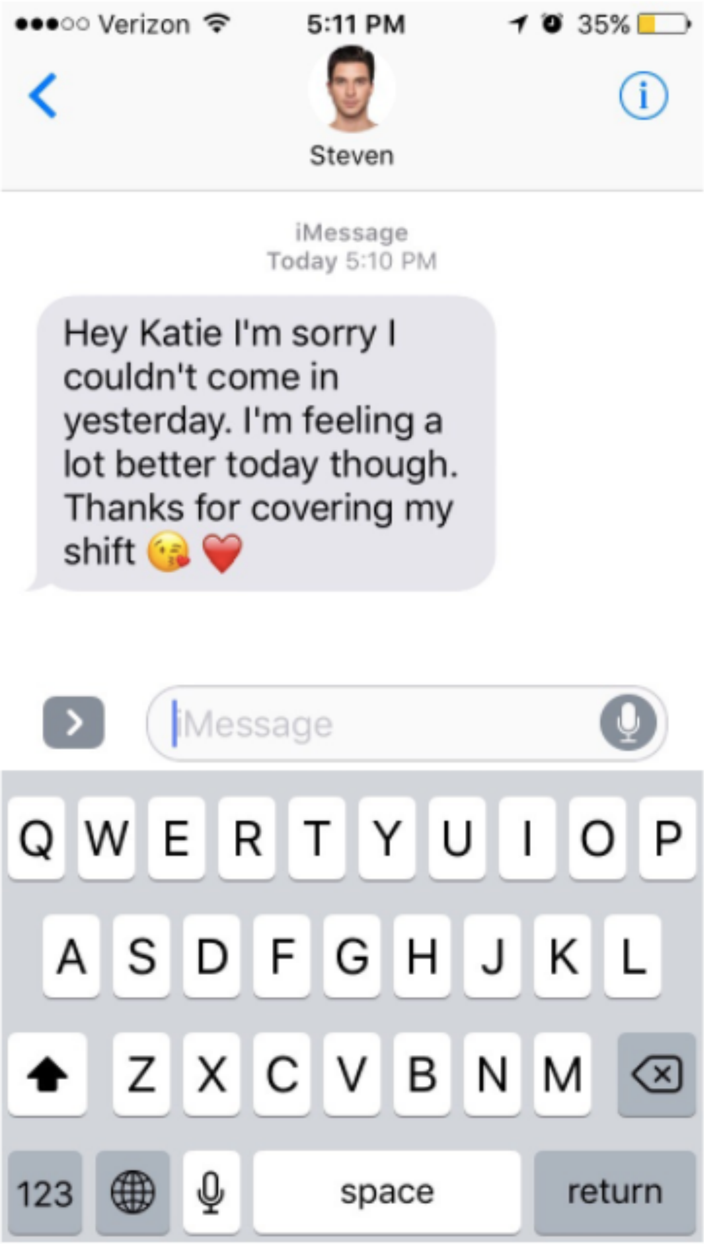

Supplement: Supplementary file 1 [file Data_Sheet_1.PDF]
